# The promise of an intersectoral network in enhancing the response to transgender survivors of sexual assault

**DOI:** 10.1371/journal.pone.0241563

**Published:** 2020-11-18

**Authors:** Janice Du Mont, Shilini Hemalal, Sarah Daisy Kosa, Lee Cameron, Sheila Macdonald

**Affiliations:** 1 Women’s College Research Institute, Women’s College Hospital, Toronto, ON, Canada; 2 Dalla Lana School of Public Health, University of Toronto, Toronto, ON, Canada; 3 Ontario Network of Sexual Assault/Domestic Violence Treatments Centres, Toronto, ON, Canada; 4 Egale Canada, Toronto, ON, Canada; University of Michigan, UNITED STATES

## Abstract

**Objectives:**

This study explores the promise of an intersectoral network in enhancing the response to transgender (trans) survivors of sexual assault.

**Methods:**

One hundred and three representatives of healthcare and community organizations across Ontario, Canada were invited to participate in a survey. Respondents were asked to: 1) identify systemic challenges to supporting trans survivors, 2) determine barriers to collaborating across sectors, and 3) indicate how an intersectoral network might address these challenges and barriers. Descriptive statistics were used to summarize quantitative data and qualitative data were collated thematically.

**Results:**

Sixty-seven representatives responded to the survey, for a response rate of 65%. Several themes capturing the challenges organizations face in supporting trans survivors were identified: Lack of knowledge and training among providers, Inadequate resources across organizations and institutions, and Limited access to and availability of appropriate services. Barriers to collaborating across sectors considered important by the overwhelming majority of respondents were: Lack of trans-positive service professionals (e.g., a paucity of sensitivity training), lack of resources (e.g., staff, staff time and workload, spaces to meet), and Institutional structures (e.g., oppressive policies, funding mandates). Four ways in which a network could address these challenges and barriers emerged from the data: Center the voices of trans communities in advocacy; Support competence of professionals to provide trans-affirming care; Provide the platform, strategies, and tools to aid in organizational change; and Create space for organizations to share ideas, goals, and resources.

**Conclusion:**

Our findings deepen our understanding of important impediments to enhancing the response to trans survivors of sexual assault and the role networks of healthcare and community organizations can play in comprehensively responding to complex health and social problems.

## Background

Persons who identify as transgender (trans), meaning those whose gender identity does not correspond with their assigned sex at birth [1, 2], experience high rates of violence, including sexual assault [3–8]. A large population-based study in the United States, the *2015 U*.*S*. *Transgender Survey* by the National Center for Transgender Equality, found that 47% of trans individuals had experienced sexual assault in their lifetime [5]. The *Sexual Violence in the Transgender Community Survey* by FORGE, an American anti-violence organization, revealed that 66% of trans persons surveyed had experienced sexual violence and 42.5% of survivors were targeted due to their gender identity [9]. In the recent cross-Canada *Trans-PULSE* survey, 26% of trans persons reported having been sexually assaulted in the previous five years [10]. Trans persons also experience high rates of poly-victimization and revictimization [2, 9, 11].

Experiences of sexual assault can have lasting impacts on the wellbeing of trans persons, contributing to physical health problems, including chronic fatigue syndrome, chronic pain, and irritable bowel syndrome; mental health conditions, such as post-traumatic stress disorder, depression, anxiety, and suicidality; and social isolation [9, 11]. For example, FORGE found that among trans survivors of sexual violence, 14% reported having experienced physical scarring as a result of their victimization, 10% long-term medical conditions, and 4% disabilities [9]. In a *Trans-PULSE* Ontario-wide survey, 29% of trans individuals had attempted suicide and 56% had seriously contemplated suicide after being sexually or physically assaulted [12]. In the same survey, almost all (97%) trans persons who had experienced an assault had avoided at least one type of public space afterwards [12].

Being subjected to sexual assault and other forms of abuse can make healing a complex process for trans survivors. However, trans persons may not have access to appropriate supports post-sexual assault, often delay in seeking needed services, or choose not to access services at all [13–16]. Obstacles to accessing services can include internal and external stigma associated with both being a sexual assault survivor *and* identifying as trans as well as with other intersecting marginalized identities, fear of being ‘outed’, fear of denial of treatment, a lack of organizational inclusive policies and practices, a lack of trust in providers, and a lack of outreach by services to trans communities [13, 14, 16]. FORGE found that only 9% of trans survivors had received professional medical care for their physical injuries and only 14% professional emotional support within the first week of being assaulted [9]. Trans survivors of sexual assault who have accessed services have reported having experienced fear, victim-blaming, unequal treatment, feeling unwelcome, verbal harassment, and culturally incompetent service providers [12, 16]. The lack of available and accessible affirming services can make trans individuals feel invisible and vulnerable [17].

The problematic response to trans survivors of sexual assault is a health equity issue that urgently needs to be addressed; it is critical that trans survivors have proper access to supports that recognize, account for, and address their diverse needs [2, 9, 14]. As no one sector alone is positioned to provide such a response [9], networks of diverse professionals and sectors might aid in achieving this goal. Networks, which foster organizational interactions of interconnected members, can bridge structural silos, highlighting shared issues and potential strategies to address them [18, 19]. Networks have been shown to improve access to resources and services; enhance practices, quality of care, and safety; and reduce stress and increase satisfaction among providers [18, 20–22].

One example of a network that has strengthened the response to trans survivors of sexual assault is the National Coalition of Anti-Violence Programs (NCAVP) in the United States, the membership of which comprises a network of 38 organizations in 22 states that “monitor, respond to, and work to end hate and domestic violence, HIV-related violence, pick-up crimes, rape, sexual assault, and other forms of violence affecting LGBTQ communities” [23], p.2. This network has worked to better engage the community, connect with youth programs, provide support for survivors, and promote trans-inclusive education and training for healthcare and community organizations. Data collected through this network have allowed for the monitoring of trends in sexual assault and domestic violence experienced by trans persons and their access to post-sexual assault services. Advocacy for the safety and support of trans survivors of violence by this large network has led to change at the local, state, and national levels. Furthermore, organizations within this network have used their enhanced competence in serving LGBTQI2S communities to provide training to other anti-violence programs [23].

Despite the numerous benefits of networks, the successful establishment of a network can be difficult. For instance, collaborators within sectors addressing violence against women have noted complications, such as power imbalances between agencies, conflicts between intervention goals, privacy concerns, lack of performance monitoring, and scarce resources [20]. Therefore, as a first step in establishing a network focused on enhancing the response to trans survivors of sexual assault in Ontario, Canada, we connected 106 representatives from 96 healthcare and community organizations identifying as trans-positive, for the purposes of learning about each other and gauging interest in working together [24]. We hosted seven regional knowledge mobilization and community engagement meetings, at which 103 of these representatives expressed ongoing interest in planning and developing a network. Discussions centered on the potential purpose and value of a network, as well as barriers and facilitators to collaborating provincially and referring reciprocally within regions [24]. The current study utilized a survey to build on critical insights arising from these discussions, which underscored the importance of assessing barriers to collaboration across sectors in a more systematic manner as well deepening our understanding of the general challenges to supporting trans survivors and the ways in which a provincial intersectoral network might contribute to addressing these challenges and barriers.

## Methods

This project was reviewed by the Women’s College Hospital Research Ethics Board (REB#2019-0073-E) and supported by an advisory group of trans community members and their allies (see Acknowledgments for advisory group membership).

### Survey

The development of the survey was guided by the advisory group and findings from the seven earlier knowledge mobilization and community engagement meetings [24]. The survey, which focused overall on next steps to forming a network [18], is described more fully in another publication [25]. It contained 37 closed- and open-ended questions organized across several domains: organizational characteristics, sociodemographic characteristics, work history, systemic challenges to supporting trans survivors, barriers to intersectoral collaboration, motivations for network membership, expectations of the network, role of the network in improving the response to trans survivors, communications strategies, and research partnerships. The survey was created in an online format in SurveyMonkey.

### Data collection

The 103 representatives of healthcare and community organizations who expressed ongoing interest in establishing a network at the knowledge mobilization and engagement meetings were invited by email in August 2019 to participate in the survey. The invitation explained that the survey was designed to gather further information related to the formation of a network to improve supports for trans survivors of sexual assault. Respondents were asked to read a letter of information and complete a consent form that preceded the survey, which addressed its purpose, potential risks and benefits of participation, and confidentiality. Three email reminders to complete the survey were sent out at one- to two-week intervals.

### Variables

Information examined in this study was respondent sociodemographic characteristics, including gender identity (choose all that apply: woman, man, bigender, trans man, trans woman, transfeminine, transmasculine, genderqueer, agender, non-binary, gender fluid, Two Spirit, other), sexual orientation (choose all that apply: lesbian, gay, bisexual, queer, Two Spirit pansexual, asexual, heterosexual, other), age group (19–24 years, 25–34 years, 35–44 years, 45–59 years, 60+ years), and ethnicity/racial background (choose one that you most identify with: Arab, West Asian, Black, Chinese, Filipino, Indigenous, Japanese, Korean, Latin American, South Asian, Southeast Asian, White, other); and work history, including role in organization (choose all that apply: support/administrative staff, coordinator, educator, frontline provider, manager/director, executive member, other), time in current role (<3 months to >10 years), time in area of employment (<3 months to >10 years), and ever having provided support services to a trans survivor of sexual assault (yes, no).

Additionally, barriers to collaborating across sectors were examined. These barriers to collaboration, which were rated on a 5-point Likert scale from 1 = not at all important to 5 = very important, were defined as: Confidentiality and privacy in making referrals and providing care across the continuum, Connecting/networking with the “right” people (e.g., high turnover of staff at organizations, lack of up-to-date contact information, lack of consistent opportunities for networking), Government/political landscape (e.g., government changes, lack of political support), Institutional structures (e.g., engagement with law enforcement, oppressive policies within the healthcare system, funding mandates of organizations), Lack of direct involvement/representation from the trans community/peer leaders at the organizational level, Lack of partnership between rural and urban regions and geographical constraints (e.g., distance, borders between organizations), Lack of resources (e.g., staff, staff time and workload, funds, compensation for peer support workers, spaces to meet), Lack of trans-positive professionals (e.g., including a paucity of sensitivity training), Lack of spaces that are considered safe and inclusive by communities when making a referral to an organization, and Siloing of sectors/organizations (e.g., community-based services shut off from healthcare or legal organizations).

Finally, we examined the systemic challenges organizations face in supporting trans survivors and how an intersectoral network might address these challenges and barriers to collaboration to enhance the response to trans survivors of sexual assault (written-in responses to open-ended questions).

### Data analysis

Survey data were exported from Survey Monkey to Microsoft Excel. Data were cleaned in Excel. Respondents who consented to participate in the survey, but did not complete any item within the survey were dropped from analysis. Frequencies and proportions were used to summarize sociodemographic and work history data. Barriers to intersectoral collaboration were described using means and standard deviations as well as frequencies and proportions were generated for each level of importance.

Written-in responses related to challenges to supporting trans survivors and how a network might address these challenges and barriers to collaboration across sectors were collated into one document for analysis. Two members of the research team independently familiarized themselves with the data and coded the text responses, including full sentences and phrases. Excerpted data with the same or very similar codes were collated into broader themes (e.g., “stigma,” “lack of trust in accessing services,” “lack of inclusive spaces,” and “availability of appropriate referrals” became the theme “Limited access to and availability of appropriate services”). To ensure themes accurately reflected respondents’ views, an additional team member reviewed them against the responses. Differences in opinion regarding the thematic categorization of data were resolved through discussion by all three team members until consensus was reached.

## Results

Sixty-seven respondents, representing 64 distinct organizations providing a diversity of services and supports to the trans community across Ontario (e.g., housing, employment, mental health, health, HIV/AIDS, Indigenous, Two Spirit, youth), filled out the survey, for a response rate of 65.0%.

### Sociodemographic characteristics

Sociodemographic characteristics of respondents are fully described in Table 1. The majority of respondents identified their gender as woman (69.2%), although two each identified as woman/trans woman/transfeminine, man/trans man/transmasculine/genderqueer/non-binary, transmasculine/non-binary, transmasculine/genderqueer/non-binary, agender/non-binary, and Two Spirit. Less than half (43.8%) of respondents indicated they were heterosexual. One quarter (25.0%) identified as queer and several others (11.0%) as queer with one or more additional orientations (e.g., two each as queer/pansexual, queer/heterosexual). Approximately a third (33.8%) of respondents were in their mid-forties to late fifties, representing the largest age group, followed by respondents who were 25 to 34 years (27.7%) and 35 to 44 years (24.6%). Respondents identified primarily as White (77.3%), but a substantial minority represented different ethnicities/racial backgrounds, including West Asian (4.5%), Black (4.5%), Indigenous (4.5%), and South Asian (4.5%).

**Table 1:** Sociodemographic characteristics.

		n	%
**Gender identity** N = 65	Woman	45	69.2
	Woman/trans woman/transfeminine	2	3.1
	Woman/genderqueer/non-binary/ genderfluid	1	1.5
	Trans woman	1	1.5
	Man	3	4.6
	Man/trans man/transmasculine	1	1.5
	Man/trans man/transmasculine/ genderqueer/non-binary	2	3.1
	Transmasculine/non-binary	2	3.1
	Transmasculine/genderqueer/non-binary	2	3.1
	Trans man	1	1.5
	Agender/non-binary	2	3.1
	Genderqueer	1	1.5
	Two Spirit	2	3.1
**Sexual orientation** N = 64	Lesbian	3	4.7
	Gay	4	6.3
	Bisexual	4	6.3
	Bisexual/queer	1	1.6
	Bisexual/queer/pansexual	1	1.6
	Queer	16	25.0
	Queer/pansexual	2	3.1
	Queer/gynosexual	1	1.6
	Queer/heterosexual	2	3.1
	Pansexual	1	1.6
	Two Spirit	1	1.6
	Heterosexual	28	43.8
**Age group** N = 65	19–24 years	6	9.2
	25–34 years	18	27.7
	35–44 years	16	24.6
	45–59 years	22	33.8
	60+ years	3	4.6
Ethnicity/racial background* N = 66	West Asian	3	4.5
	Black	3	4.5
	Filipino	1	1.5
	Indigenous	3	4.5
	Korean	1	1.5
	South Asian	3	4.5
	White	51	77.3
	Biracial (Arabic & White)	1	1.5

*Ethnicity/racial background that respondent most identifies with

### Work history

The work history of respondents is fully described in Table 2. Most respondents were frontline providers (50.8%), such as clinicians, nurses, and counsellors. Many respondents were also coordinators (43.1%), educators (26.2%), or managers/directors (24.6%) in the organizations they represented. Respondents were experienced; almost two-fifths (38.5%) had worked in their current role for three or more years. More than half (53.1%) had been engaged in this area of employment for more than ten years. Nearly three quarters (73.8%) indicated that they had experience providing support services to a trans sexual assault survivor.

**Table 2:** Work history.

		n	%
Role in organization* N = 65	Support/administrative staff	3	4.6
	Coordinator	28	43.1
	Educator	17	26.2
	Frontline provider	33	50.8
	Manager/director	16	24.6
	Executive member	2	3.1
	Volunteer	1	1.5
**Time in current role** N = 65	0 to <3 months	5	7.7
	3 months to <6 months	5	7.7
	6 months to <1 year	5	7.7
	1 year to <3 years	25	38.5
	3 years to <5 years	8	12.3
	5 years to <10 years	7	10.8
	10+ years	10	15.4
**Time in area of employment** N = 64	1 year to <3 years	14	21.9
	3 years to <5 years	6	9.4
	5 years to <10 years	10	15.6
	10+ years	34	53.1
**Ever provided support services to a trans survivor of sexual assault** N = 65	Yes	48	73.8
	No	14	21.5
	Not applicable	3	4.6

*Categories are not mutually exclusive

### Challenges to supporting trans survivors of sexual assault

**Fig 1:**
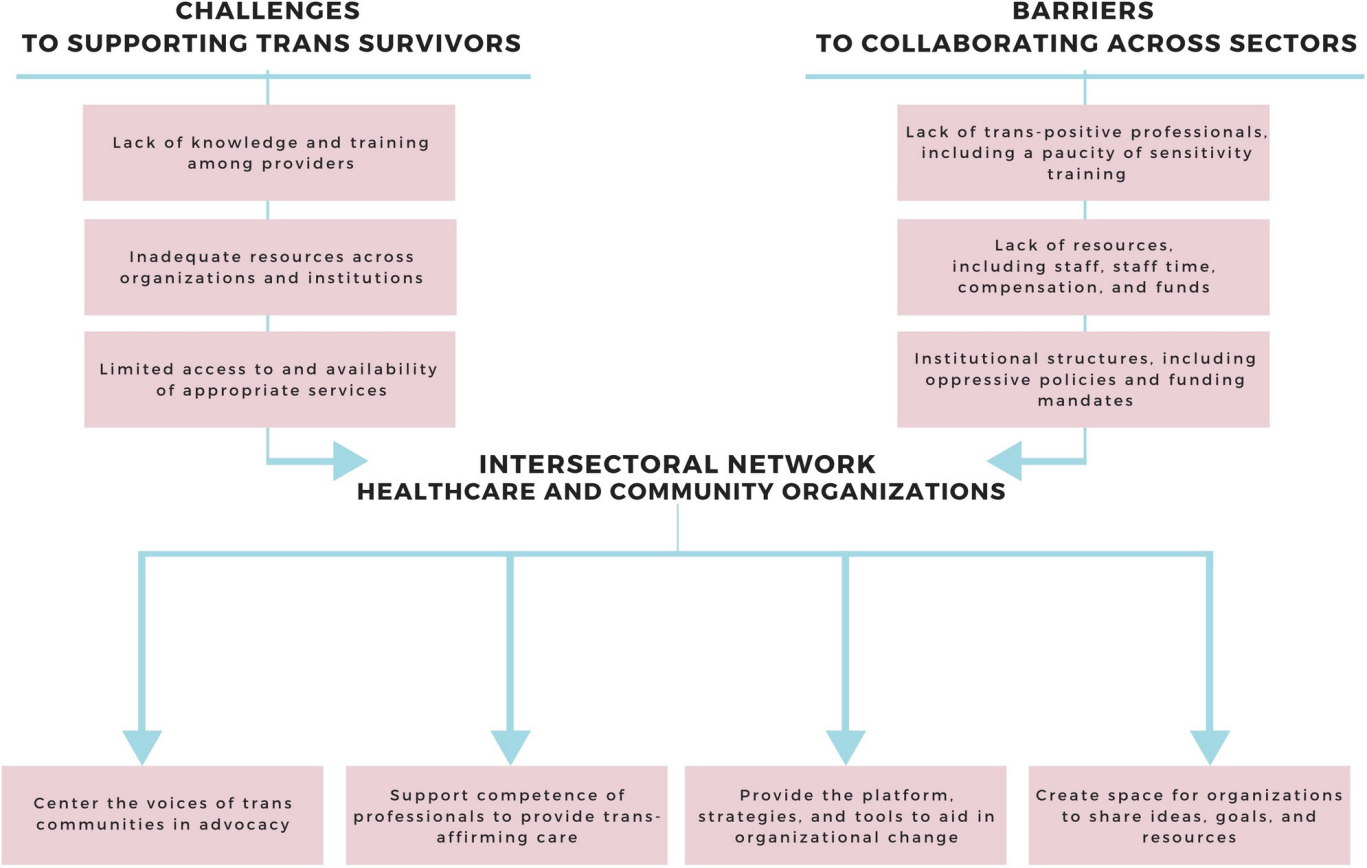
The promise of an intersectoral network in addressing challenges and barriers to enhancing the response to trans survivors of sexual assault.

Three themes captured the systemic challenges respondents described their organizations face in providing support services for trans survivors (Fig 1): Lack of knowledge and training among providers, Inadequate resources across organizations and institutions, and Limited access to and availability of appropriate services.

#### Lack of knowledge and training among providers

A “[l]ack of awareness… on the part of healthcare providers to trans specific issues” was identified by respondents as a significant impediment to supporting trans sexual assault survivors *(Director*, *Central region healthcare organization)*. A coordinator and frontline provider in a Southwest region healthcare organization stated that in this context, there is “limited education around … sexual assault and domestic violence;” a sentiment echoed by a representative of a community organization who noted a “[l]ack of specific training on how to provide services to trans clients who have survived sexual assault” *(Frontline Provider*, *Central West region)*. A manager of a Southwest region community organization further observed that there is a “lack of knowledge on identities and barriers faced by [the trans] community”. Other gaps in knowledge respondents perceived as affecting their ability to support survivors were: “[use of] proper language [when] serving trans population[s]” *(Frontline Provider*, *Southwest region healthcare organization)* and “knowledge of other service providers and institutions'' *(Manager and Educator*, *Southwest region community organization)*. A problem revealed in relation to a “[l]ack of formal training” was the “[l]ow volume of cases to develop competency” *(Manager*, *East region healthcare organization)*.

#### Inadequate resources across organizations and institutions

Respondents stated that a scarcity of various types of resources materially impacted their ability to help trans survivors, citing specifically, being “underfunded/under supported” (*Support Staff*, *Coordinator*, *Educator*, *and Frontline Provider*, *Central region community organization)*. Limited knowledge and training about the care of trans survivors was associated, for instance, with a lack of “[r]esources for… devoted trained staff” (*Executive Member*, *Southwest region community organization)*. Respondents observed, “[they]struggle to offer timely services as [they] often have… long waitlist[s] due to staffing shortages” *(Manager*, *Central region community organization*). A support staff, coordinator, educator, and frontline provider in a Central region community organization also alluded to the fact that there are not always sufficient resources to provide individualized supports to “nonbinary, transgender, and two spirit people … experienc[ing] sexualized violence,” noting “I can occasionally accompany them to [legal] appointments.” Some respondents indicated that their organizations had challenges obtaining resources to serve trans persons at all. The director of a Central East region community organization commented that they are funded only to provide services for women, meaning that they have to “[o]btain other forms of funding to provide services to people of all genders.”

#### Limited access to and availability of appropriate services

Respondents remarked that it can be difficult to support trans survivors because there is a dearth of “[i]nclusive spaces that trans folks can access without facing discrimination” *(Educator*, *Northeast region community organization)*. In this regard, it was observed, in particular, that there is a “[l]ack of trust in accessing healthcare services by the trans community” (*Director*, *Central region healthcare organization*). Respondents acknowledged that trans persons face both the “stigma” associated with their trans identity and being a sexual assault survivor, which can result in a “[l]ack of willingness to come forward in identifying as a survivor of sexual assault” *(Educator and Frontline Provider*, *Central East region community organization)* and be further exacerbated by other marginalizing “issues of racism, poverty, social location, etc.” *(Manager*, *Northwest region healthcare organization)*. A manager of a healthcare organization working with rural and remote communities in the Northwest region of the province related:

“[D]ue to the geography of our area, the systemic challenges faced by trans clients are linked to the same systemic challenges around access to timely, equitable and ongoing services (across the health/wellness continuum) that all our First Nations and northern clients face.”

Respondents also noted that there were problems with the availability of trans-positive services to which they could make referrals. One frontline provider from a Central region healthcare organization stated, “[r]eferral resources for trans youth who have experienced sexual abuse/assault/exploitation are limited,” elaborating “[i]t is difficult to find community mental health agencies that we feel comfortable referring to.” Even in circumstances in which respondents perceived services to be available, they were sometimes described as unsuitable due to their non-inclusive infrastructure design or delivery, particularly within the healthcare sector:

“Preferred names have recently been added to charts, but preferred pronouns and gender are not readily available without searching through the chart. Lack of trans-friendly signage and pamphlets.” (*Frontline Provider*, *East region healthcare organization]*

“[Need for a]daption of … computer charting system to ensure proper identification.”*(Manager*, *Central East region healthcare organization)*

### Barriers to collaboration across sectors

Three barriers to collaborating across sectors were identified as important/very important by the overwhelming majority of respondents (Fig 1): Lack of trans-positive professionals, including a paucity of sensitivity training (94.1%); Lack of resources, including staff, staff time and workload, funds, compensation for peer support workers, and spaces to meet (92.2%), and Institutional structures, including engagement with law enforcement, oppressive policies within the healthcare system, and funding mandates of organizations (88.2%). See S1 Table for a full listing of barriers and associated frequencies, proportions, means, and standard deviations.

### Potential of an intersectoral network to enhance the response to trans survivors of sexual assault

Four themes captured how respondents thought that a provincial intersectoral network could address challenges and barriers to improving the response to trans survivors (Fig 1): Center the voices of trans communities in advocacy; Support competence of professionals to provide trans-affirming care; Provide the platform, strategies, and tools to aid in organizational change; and Create space for organizations to share ideas, goals, and resources.

#### Center the voices of trans communities in advocacy

Respondents observed that a crucial function of any network in improving the response to trans survivors would be advocacy. A director of a Central region community organization stated that a network could “[p]rovid[e] advocacy in various areas (macro and mezzo).” In particular, respondents suggested, a network could address the issue of “funding transparency,” “[c]urrent working understandings of evidence-based funding [that] historically exclude unrecognized populations,” “[the] public/private healthcare divide” (*Coordinator*, *Central West region community organization*), and the “[provincial health insurance plan’s] back facing retained gender” (*Support Staff*, *Coordinator*, *Educator*, *and Frontline Provider*, *Central region community organization)*. Additionally, it was proposed that “doing advocacy within [community-based violence agencies and h]iring… nonbinary/trans advocates/caseworkers to accompany survivors to appointments” could improve supports available to trans survivors (*Support Staff*, *Coordinator*, *Educator*, *and Frontline Provider*, *Central region community organization)*. Respondents clearly articulated that the voices of trans persons would need to be centered in all advocacy work:

“It is important to engage with community members, service providers, policy makers, and political groups simultaneously. Trans, Non-Binary, and Two-Spirit individuals need to be a part of the decision making, consultation, and implementation processes, with respect to race, age, sexual orientation, disability, immigration status, and socio-economic circumstance, as these factors further marginalize the voices of Trans, Non-Binary and Two-Spirit folks.” *(Coordinator*, *Central region community organization)*

#### Support competence of professionals to provide trans-affirming care

Respondents commented on the important role that a network could play in enhancing the response to sexual assault of trans persons by providing a “[f]ree mandatory centralized body of education” (*Coordinator*, *Central West region community organization)*, focused on “best practices and delivery of services” *(Frontline Provider*, *Southwest region healthcare organization)* and “nonbinary and trans survivor[‘s] experiences and needs (AFAB and AMAB)” (*Support Staff*, *Coordinator*, *Educator*, *and Frontline Provider*, *Central region community organization)*. A coordinator, educator, and frontline provider in a Southwest region healthcare organization noted that educational efforts within a network could “[c]ontinue bringing different sectors together to learn and grow… [and p]rovide ongoing learning opportunities that are current in a variety of educational formats,” fostering learning that is both reciprocal and interactive. This was a view reiterated by a manager of an East region healthcare organization who stated, members of a network could “[p]rovide support and offer to teach … trainings through interactive learning rather than just online.” The importance of training professionals was underscored by respondents particularly in the healthcare context, within which, it was observed, the “[d]evelopment of a network could support [those] providing frontline care [by offering] regional meetings/training” (*Manager*, *East region healthcare organization)*. One coordinator of a Central West region community organization also indicated that a network could “[aid in the] integration of trans healthcare into standardized medical curricula.”

#### Provide the platform, strategies, and tools to aid in organizational change

Respondents remarked that a network could address the challenges and barriers to providing quality care to trans survivors by “[s]upport[ing] organizations as they work on new policies/procedures and ways of thinking that are inclusive to all” (*Coordinator*, *Educator*, *and Frontline Provider*, *Southwest region healthcare organization)*. A network could, it was observed, “[h]elp programs develop and align plans for implementation at a strategic level” (*Manager*, *East region healthcare organization)* and address “ideological biases at [the] policy level” *(Coordinator*, *Central West region community organization)*. A frontline provider in an East region healthcare organization suggested that a network could even improve the technological infrastructure and culture of hospitals by recommending new collaborators, such as *“*tech companies that [could] develop [appropriate] EMRs [electronic medical records]”, and “providing [trans-inclusive] posters and pamphlets.”

#### Create space for organizations to share ideas, goals, and resources

Respondents asserted that a network could address the myriad of obstacles in the way of better supporting trans survivors by “[p]roviding a province-wide forum for organizations to connect” *(Educator and Frontline Provider*, *Central region healthcare organization)*. An educator and frontline provider from a Northeast community organization noted, for example, that a network could focus on “[h]osting conference[s], meetings, and workshops bringing all frontline workers [in the community] and those in healthcare related fields together to discuss visions.” One coordinator of a Central region community organization further explained that a network could have a role in “[f]ostering … collaboration, pathway mapping, promoting ground-level education and population-level research.” Establishment of a network was also viewed as a viable mechanism through which to share existing knowledge, a director of a Central East community organization indicating, “[t]here is research about [their] local hospital and health services regarding trans-inclusion” and offering to “connect [the researchers] to that important research in [their] region.” Respondents also observed that a network could facilitate reciprocal referrals and strengthen partnerships among organizations by “[e]stablishing a list of available resources, support services, and organizations in each area” (*Educator and Frontline Provider*, *East region healthcare organization*) and facilitating “regional subgroups to coordinate care close to home” *(Educator and Frontline Provider*, *Central region healthcare organization)*. This could include, respondents proposed, promoting “communicati[on] between organizations, trans communities, and healthcare networks as a continuous and ongoing conversation” *(Coordinator*, *Central West region community organization*).

## Discussion

The sexual assault of trans persons is an insidious problem, with trans persons experiencing high rates of victimization and serious psychosocial and health consequences that no single discipline or sector has the training or resources to address [4, 6, 8, 9, 11]. Our study further lays the foundation for an Ontario-wide intersectoral network of healthcare and community organizations that holds the potential to substantially enhance the response to trans survivors of sexual assault [24, 25]. Sixty-seven experienced representatives from 64 distinct and diverse organizations from across the province identified important challenges to supporting trans survivors, as well as barriers to being able to work together effectively. However, they concurrently recognized and made suggestions for how a network could facilitate connections across sectors and better address the needs of trans survivors. These findings improve our understanding of the role that networks can play in comprehensively responding to complex health and social problems [26, 27].

A lack of knowledge and training among providers on how to provide trans-specific care, including the language to use in supporting survivors, was identified by representatives of participating organizations as a systemic challenge to supporting trans sexual assault survivors. This finding is congruent with results rating a lack of service professionals who are trans-positive as the most significant barrier to collaborating across sectors to enhance care. It is also consistent with several previous studies that have identified a lack of training, limited medical knowledge, and scant access to appropriate education among healthcare and social service providers results in inadequate care and experiences of discrimination when trans persons seek supports [13, 14, 16, 28–30]. Organizational representatives indicated that a network could address these problems by supporting providers in becoming more knowledgeable about trans issues and experiences while working to improve their competence to provide trans-affirming care. A network could build this capacity, it was proposed, for example, by developing tangible educational tools and reciprocal in-person trainings to promote up-to-date, appropriate, and sensitive supports for trans persons. Cross-sectoral training has been found to be a successful approach in promoting intersectoral collaboration in other studies [31] and, furthermore, was identified as an important need in the earlier planning stage of the network [24].

Inadequate resources across organizations and institutions, including a lack of funding and supports, was identified by surveyed representatives as a significant challenge to appropriately serving trans survivors. This finding supports their determination also that a lack of resources, such as staff time and workload, is an important barrier to working together to provide trans-affirming care. Resource limitations have been acknowledged as an impediment to intersectoral collaboration and the delivery of first-rate care both in previous stages of the development of this network and other studies [32, 33]. For example, a survey of community-based rape crisis and sexual assault centers found that underfunding and reliance on volunteers limited their capacity fully to support survivors [34]. As was also noted by organizational representatives in our study, Beres and colleagues further indicated that some centers and programs serve women only in accordance with funding policies [34]. However, these representatives thought that a network, which centers the voices, expertise, and needs of trans communities, could mobilize advocacy efforts to address funding models that exclude “unrecognized populations”, as well assist more generally with funding and staffing issues that impact responses to trans sexual assault survivors, as is supported in other literature [24, 35, 36].

Limited access to and availability of appropriate services were identified by representatives of organizations as substantial challenges to the response to trans survivors. As in earlier studies, they associated discrimination and stigma related to being trans *and* a sexual assault survivor to a lack of trust in accessing healthcare services [13, 14, 16]. It was mentioned that these experiences intersect with other forms of oppression, including racism and poverty, which further limit access to supports; findings similar to those in research on trans persons experiencing sexual assault and homelessness [37]. Additionally, a shortage of trans-positive services was highlighted [23, 38], with infrastructure design and delivery identified as a particularly problematic issue in the healthcare system (issues with the environment and information collection systems). Earlier studies of trans health similarly have found a lack of inclusive signage, electronic health records, documentation, billing/coding systems, and laboratory information systems results in care that excludes trans persons from the outset [2, 35]. Not surprisingly then, problematic institutional structures also were perceived to be a significant barrier to intersectoral collaboration. Nonetheless, with respect to these issues, representatives stated that a network could support organizational change by providing the necessary platform, strategies, tools, and space to share ideas, goals, and resources, leading to improved inclusiveness of services.

### Limitations and strengths of the study

This study has limitations that are important to acknowledge. Although we had a robust response to our survey (65.0%), the results may be subject to selection bias. Representatives from healthcare and community organizations who filled out the survey may hold different opinions than those who did not. Also, while the insights gleaned in this study related to systemic challenges to collaborating and supporting trans survivors could have applicability to other jurisdictions seeking to improve their response to sexual assault, access to and availability of services and supports dedicated to or inclusive of trans persons vary widely across the globe.

A key strength of this study was the level of experience and diversity of participating organizational representatives. More than half were frontline providers and had over a decade of experience working in the area; almost three quarters had provided sexual assault support services to a person identifying as trans. While there is a historical lack of gender diverse persons represented in research [39], more than one quarter of respondents in our study did not identify solely as a woman or man, providing their unique lived experiences to identifying challenges and barriers to enhancing trans sexual assault supports. Also of note, those who did identify as trans, often did not identify solely as trans; for example, one respondent identified as man/trans man/transmasculine/genderqueer/non-binary. Similarly, when indicating their sexual orientation, several respondents who selected queer, chose additional orientations that provided more specificity (e.g., queer/gynosexual). These findings are important to consider in the design of future research studies as they demonstrate that data collection tools that do not allow for fluidity or a full range of options within gender identity and sexual orientation questions may be inherently exclusive of the diversity within LGBTQI2S+ populations [1].

## Conclusion

Our study contributes to a better understanding of the challenges that healthcare and community organizations may face in providing supports to trans survivors, as well as the factors that hinder collaborating across sectors to enhance the response to sexual assault. Representatives of diverse organizations identified several important ways in which a provincial intersectoral network could address these obstacles to improved care—a small step in answering the global call for equity for trans persons [40]. In future research, we will work with healthcare and community collaborators and a peer leadership group to focus and expand the network [18, 24, 25]. It will be critical to evaluate the ability of the network to enhance the response to trans survivors of sexual assault at later stages in its establishment, using metrics that reflect changes in the challenges to providing appropriate supports and barriers to intersectoral collaboration identified in this study.

## Supporting information

S1 Table Barriers to collaboration across sectors. (DOCX)
