# Supplementary material for: The promise of an intersectoral network in enhancing the response to transgender survivors of sexual assault
Source: PLoS One. 2020 Nov 18;15(11):e0241563. doi: 10.1371/journal.pone.0241563 (PMC7673577; doi:10.1371/journal.pone.0241563)
Supplement: S1 Table — (DOCX) [file pone.0241563.s001.docx]

**S1 Table. Barriers to collaboration across sectors**

| **Barrier** | **Rating** | **n** | **%** | **Mean** | **SD** |
| --- | --- | --- | --- | --- | --- |
| Lack of trans-positive professionals (e.g., a paucity of sensitivity training)  N=51 | Not at all important | 1 | 2.0 | 4.5 | 0.81 |
|  | Somewhat unimportant | 1 | 2.0 |  |  |
|  | Neither unimportant nor important | 1 | 2.0 |  |  |
|  | Important | 15 | 29.4 |  |  |
|  | Very important | 33 | 64.7 |  |  |
| Lack of resources (e.g., staff, staff time and workload, funds, compensation for peer support workers, spaces to meet)  N=51 | Not at all important | 0 | 0.0 | 4.3 | 0.79 |
|  | Somewhat unimportant | 3 | 5.9 |  |  |
|  | Neither unimportant nor important | 1 | 2.0 |  |  |
|  | Important | 23 | 45.1 |  |  |
|  | Very important | 24 | 47.1 |  |  |
| Institutional structures (e.g., engagement with law enforcement, oppressive policies within the healthcare system, funding mandates of organizations)  N=51 | Not at all important | 1 | 2.0 | 4.3 | 0.89 |
|  | Somewhat unimportant | 2 | 3.9 |  |  |
|  | Neither unimportant nor important | 3 | 5.9 |  |  |
|  | Important | 22 | 43.1 |  |  |
|  | Very important | 23 | 45.1 |  |  |
| Lack of direct involvement/representation from the trans community/peer leaders at the organizational level  N=50 | Not at all important | 0 | 0.0 | 4.2 | 0.95 |
|  | Somewhat unimportant | 5 | 10.0 |  |  |
|  | Neither unimportant nor important | 3 | 6.0 |  |  |
|  | Important | 19 | 38.0 |  |  |
|  | Very important | 23 | 46.0 |  |  |
| Lack of spaces that are considered safe and inclusive by communities when making a referral to an organization  N=51 | Not at all important | 2 | 3.9 | 4.2 | 1.07 |
|  | Somewhat unimportant | 3 | 5.9 |  |  |
|  | Neither unimportant nor important | 4 | 7.8 |  |  |
|  | Important | 18 | 35.3 |  |  |
|  | Very important | 24 | 47.1 |  |  |
| Lack of partnership between rural and urban regions and geographical constraints (e.g., distance, borders between organizations)  N=51 | Not at all important | 0 | 0.0 | 4.1 | 0.89 |
|  | Somewhat unimportant | 4 | 7.8 |  |  |
|  | Neither unimportant nor important | 5 | 9.8 |  |  |
|  | Important | 22 | 43.1 |  |  |
|  | Very important | 20 | 39.2 |  |  |
| Siloing of sectors/organizations (e.g., community-based services shut off from healthcare or legal organizations)  N=51 | Not at all important | 0 | 0.0 | 4.1 | 0.80 |
|  | Somewhat unimportant | 2 | 3.9 |  |  |
|  | Neither unimportant nor important | 8 | 15.7 |  |  |
|  | Important | 25 | 49.0 |  |  |
|  | Very important | 16 | 31.4 |  |  |
| Government/ political landscape (e.g., government changes, lack of political support)  N= 51 | Not at all important | 0 | 0.0 | 4.0 | 0.93 |
|  | Somewhat unimportant | 6 | 11.8 |  |  |
|  | Neither unimportant nor important | 3 | 5.9 |  |  |
|  | Important | 26 | 51.0 |  |  |
|  | Very important | 16 | 31.4 |  |  |
| Connecting/networking with the “right” people (e.g., high turnover of staff at organizations, lack of up-to-date contact information, lack of consistent opportunities for networking)  N=51 | Not at all important | 1 | 2.0 | 4.0 | 1.02 |
|  | Somewhat unimportant | 5 | 9.8 |  |  |
|  | Neither unimportant nor important | 5 | 9.8 |  |  |
|  | Important | 22 | 43.1 |  |  |
|  | Very important | 18 | 35.3 |  |  |
| Confidentiality and privacy in making referrals and providing care across the continuum  N=51 | Not at all important | 1 | 2.0 | 3.8 | 1.11 |
|  | Somewhat unimportant | 7 | 13.7 |  |  |
|  | Neither unimportant nor important | 9 | 17.6 |  |  |
|  | Important | 17 | 33.3 |  |  |
|  | Very important | 17 | 33.3 |  |  |
